# Creatinine-based GFR-estimating equations in children with overweight and obesity

**DOI:** 10.1007/s00467-021-05396-y

**Published:** 2022-02-24

**Authors:** Mark J. C. M. van Dam, Hans Pottel, Anita C. E. Vreugdenhil

**Affiliations:** 1 Centre for Overweight Adolescent and Children’s Healthcare (COACH), Department of Pediatrics, School of Nutrition and Translational Research in Metabolism (NUTRIM), Maastricht University Medical Centre +, P. Debyelaan 25, 6229 HX Maastricht, The Netherlands; 2 Department of Public Health and Primary Care, KU Leuven Campus Kulak Kortrijk, Kortrijk, Belgium

**Keywords:** Childhood obesity, Creatinine, eGFR, Pediatrics

## Abstract

**Background:**

With the increasing prevalence of childhood obesity and related development of chronic kidney disease (CKD), there is a critical need to understand how best to assess kidney function in children with obesity. Since serum creatinine (SCr) is recommended as marker of first choice for GFR estimation, we evaluated and compared creatinine-based GFR equations in children with overweight and obesity.

**Methods:**

Six hundred children with overweight and obesity (53.5% female; mean age 12.20 ± 3.28 years; mean BMI z-score 3.31 ± 0.75) were included from the Centre for Overweight Adolescent and Children’s Healthcare (COACH).

**Results:**

Serum creatinine (SCr), normalized using Q-age polynomials obtained from reference values, results in median and mean SCr/Q value close to “1” for all age groups, and 96.5% of the children have a SCr/Q within the reference band [0.67–1.33], corresponding to the 2.5th and 97.5th percentile. eGFR CKiD (bedside Schwartz equation) and Schwartz-Lyon decreased with age, whereas eGFR EKFC and modified CKD-EPI40 showed no age-dependency, but the distribution of eGFR values was not symmetrical. eGFR CKiD under 25 (CKiDU25) demonstrated no age-dependency but major sex differences were observed. eGFR FAS age, FAS height, and adjusted-creatinine revised Lund-Malmö (LMR18) showed a relatively symmetrical distribution and no age-dependency.

**Conclusions:**

Serum creatinine (SCr) values of children with overweight and obesity are mostly within the reference range for children. Normalization of SCr using reference Q-age polynomials works very well in this cohort. After evaluation of the different equations, we suggest that FAS age, FAS height, and LMR18 are the preferred creatinine-based GFR-estimating equations in children with overweight and obesity.

Clinical trial registration.

ClinicalTrial.gov; Registration Number: NCT02091544.

**Graphical abstract:**

A higher resolution version of the Graphical abstract is available as Supplementary information
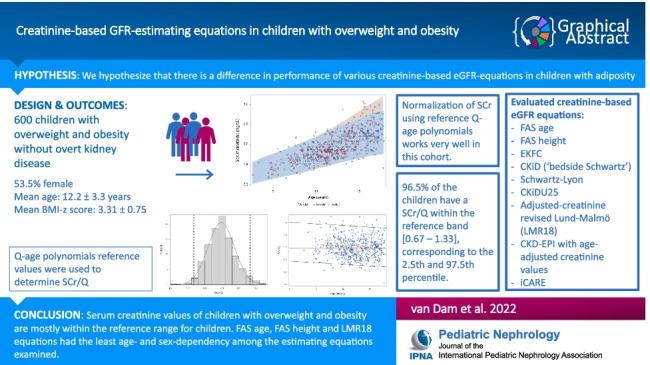

**Supplementary Information:**

The online version contains supplementary material available at 10.1007/s00467-021-05396-y.

## Introduction

Glomerular filtration rate (GFR) estimation is essential for daily practice in (pediatric) nephrology, since direct measurement of GFR is still considered to be too invasive for routine clinical use. Estimated GFR (eGFR) equations are highly dependent on the data used for the development of the equation and incorrect use of an equation might lead to severe inaccuracy [1]. In adults with overweight and obesity, GFR-estimating equations fail in their precision to estimate GFR [2]. Given the increasing prevalence of childhood obesity, there is a need to obtain more knowledge on how best to estimate GFR in this population, both concerning research and clinical questions such as drug dosing or chronic kidney disease (CKD) classification.

GFR-estimating equations using endogenous filtration markers (such as serum creatinine (SCr) and cystatin C) are suffering from inaccuracy (bias) and imprecision (random error). When using these equations, physicians should be aware of the factors besides the GFR that influence serum concentrations of endogenous filtration markers (the “non-GFR determinants”) such as dietary intake, synthesis, tubular absorption and secretion, and kidney-independent elimination [3]. In order to partly correct for these non-GFR determinants, demographic and clinical variables such as age, sex, height, and body surface area (BSA) are implemented in eGFR equations. However, these factors do not fully encompass the altered body proportions in children and adults with adiposity. Moreover, most commonly used eGFR equations were developed and validated in individuals with predominantly normal body composition [4].

Although other endogenous filtration markers are available, SCr is still recommended as marker of first choice for GFR evaluation. Creatinine, synthesized in the liver and kidneys, is stored predominantly in striated muscle cells [5]. The volume of distribution of creatinine is total body water [6] and it is known that SCr is influenced by lean body mass [7]. Besides endogenous creatinine production, dietary protein intake and creatine supplements may contribute to the serum creatinine concentration [3]. In children, SCr linearly increases with age between the age of 2 and 14 years and levels off to constant values of 0.90 mg/dL for adult males and 0.70 mg/dL for adult females. GFR is indexed with BSA, allowing comparison among children and adults [1, 8–11]. Although indexation of GFR for BSA for individuals with altered body proportions is a controversial topic [2, 12, 13], almost all current creatinine-based GFR-estimating equations have been designed for GFR indexed with BSA [1, 4].

The association between obesity in adulthood and incidence of CKD and CKD progression is currently well established [14] and there is some evidence of an association between childhood obesity and CKD later in life [15]. This, combined with the increasing prevalence of childhood obesity, underlines the need to understand how best to assess kidney function in children with obesity. Therefore, we evaluated SCr and creatinine-based GFR-estimating equations in children with overweight and obesity.

## Methods

### Setting and study inclusion

For this study, baseline data were used from participants of the Centre for Overweight Adolescent and Children’s Healthcare (COACH) at the Maastricht University Medical Centre + (MUMC +). COACH is a multidisciplinary lifestyle program in which the health status of children with overweight and obesity is evaluated, as described previously [16]. Children who enter the COACH program are evaluated thoroughly in order to examine possible secondary causes of overweight and obesity and obesity-associated comorbidities. This first evaluation includes history taking, clinical examination and laboratory tests among others. All 662 participants who entered the COACH program between January 1, 2011, and April 1, 2019, were considered for inclusion in this study. Children with secondary causes of overweight, a history of (congenital or acquired) kidney disease that was known before inclusion into our program, diabetes mellitus, and/or current use of antihypertensive medication were excluded from this study (*n* = 13). Moreover, in 39 children the value for serum creatinine (SCr) was missing. Five children did not meet the criteria for overweight or obesity and 5 children were older than 18 years and were thus excluded from this study. Overall, 600 children were included in this study. The study met the guidelines administered by the Declaration of Helsinki and was approved by the Medical Ethical Committee of the MUMC + . Informed consent was obtained from all parents or legal guardians and children, provided they were 12 years or older.

### Clinical assessment and anthropometry

All children underwent a physical examination. Weight and height were determined using a digital scale (Seca, Chino, CA) and digital stadiometer (De Grood Metaaltechniek, Nijmegen, the Netherlands), respectively. The International Obesity Task Force (IOTF) criteria were used to define overweight, obesity, and severe obesity [17]. Body surface area (BSA) was calculated using height (cm) and weight (kg), using the Haycock et al. equation: (*Ht*) ($BSA=0.024265\times {Wt}^{0.5378}\times {Ht}^{0.3964}$) [18].

### Creatinine-based GFR-estimating equations

Serum creatinine (SCr) was measured using the enzymatic method (Cobas 8000, Roche), equivalent to the isotope dilution mass spectrometry (IDMS) gold standard method. Creatinine references values (median SCr for “healthy children,” so-called Q-values) were obtained from the literature [19, 20]. These Q-values were used to calculate SCr/Q (“normalized SCr”) which is supposed to be age- and sex-independent for healthy subjects. It has been shown by Pottel et al. [21] that the reference band for SCr/Q in children is defined by [0.67–1.33], with lower and upper limits corresponding to the 2.5th and 97.5th percentile. GFR was estimated using the following creatinine-based GFR-estimating equations, displayed in Table 1:

- Height-independent full-age spectrum equation, referred to as FAS age [22];
- Height-dependent full-age spectrum equation, referred to as FAS height [20];
- The new European Kidney Function Consortium (EKFC) equation, referred to as EKFC [23];
- Updated bedside Schwartz or CKiD equation [24];
- Schwartz-Lyon equation [25];
- CKiD under 25 years equation (CKiDU25) [26];
- Adjusted-creatinine revised Lund-Malmö equation, referred to as LMR18 [27];
- CKD-EPI equation with age-adjusted creatinine values, referred to as CKD-EPI40 [28];
- Improving renal complications in Adolescents with T2D through Research equation, referred to as iCARE [29].

**Table 1:** Overview of the creatinine-based GFR-estimating equations examined in this study

FAS age [22]	$eGFR=107.3/{~}^{(SCr}\!\left/ \!\!{~}_{{Q}_{age}}\right.)$, in which ${Q}_{age}=0.21+0.057\times Age-0.0075\times {Age}^{2}+0.00064\times {Age}^{3}-0.000016\times {Age}^{4}$ for males and ${Q}_{age}=0.23+0.034\times Age-0.0018\times {Age}^{2}+0.00017\times {Age}^{3}-0.0000051\times {Age}^{4}$ for females
FAS height [20]	$eGFR=107.3/{~}^{(SCr}\!\left/ \!\!{~}_{{Q}_{height})}\right.$, in which ${Q}_{height}=3.94-13.4\times L+17.6\times {L}^{2}-9.84\times {L}^{3}+2.04\times {L}^{4}$, in which *L* represents height in meters for males and females
EKFC [23]	$eGFR=107.3/({{~}^{SCr}\!\left/ \!\!{~}_{Q}\right.)}^{A}[\times {0.990}^{Age-40} \text{if} age>40 \text{years}]$ with *A* = + 0.322 when *SCr*/*Q* < 1 and *A* = + 1.132 when *SCr*/*Q* ≥ 1, in which *Q* (in µmol/L) is obtained from $\mathit{ln}\left(Q\right)=3.200+0.259\times Age-0.543\times \mathit{ln}\left(Age\right)-0.00763\times {Age}^{2}+0.0000790\times {Age}^{3}$ for males and $\mathit{ln}\left(Q\right)=3.080+0.177\times Age-0.223\times \mathit{ln}\left(Age\right)-0.00596\times {Age}^{2}+0.0000686\times {Age}^{3}$ for females, when 2 ≤ *Age* ≤ 25 years
CKiD (“bedside Schwartz”) [24]	$eGFR=k\times {~}^{L}\!\left/ \!\!{~}_{SCr}\right.$ in which *k* is 0.413, *L* represents height in cm, and *SCr* is expressed in mg/dL
Schwartz-Lyon [25]	$eGFR=k\times {~}^{L}\!\left/ \!\!{~}_{SCr}\right.$ in which *k* is 0.413 if males age > 13 years and *k* is 0.368 otherwise, *L* represents height in cm, and *SCr* is expressed in mg/dL
CKiDU25 [26]	$eGFR=k\times {~}^{L}\!\left/ \!\!{~}_{SCr}\right.$ in which for males $k=39.0\times {1.008}^{\left(age-12\right)}$ for 1 ≤ age < 12, $k=39.0\times {1.045}^{\left(age-12\right)}$ for 12 < age < 18, and $k=50.8$ for 18 ≤ age < 25; for females $k=36.1\times {1.008}^{\left(age-12\right)}$ for 1 ≤ age < 12, $k=36.1\times {1.023}^{\left(age-12\right)}$ for 12 < age < 18, and $k=41.4$ for 18 ≤ age < 25. *L* represents height in meters and *SCr* is expressed in mg/dL
LMR18 [27]	$eGFR={e}^{X-0.0158\times \mathit{max}\left(Age;18\right)+0.438\times ln(max\left(Age;18\right))}$ in which $X=2.50+0.0121\times (150-Q)$ for females with *Q* < 150 µmol/L, $X=2.50-0.926\times ln({~}^{Q}\!\left/ \!\!{~}_{150}\right.)$ for females with *Q* ≥ 150 µmol/L, $X=2.56+0.00968\times (180-Q)$ for males with *Q* < 180 µmol/L, and $X=2.56-0.926\times ln({~}^{Q}\!\left/ \!\!{~}_{180}\right.)$ for males with *Q* ≥ 180 µmol/L, in which *Q* is obtained from $\mathit{ln}\left(Q\right)=\mathit{ln}\left(SCr\right)+0.259\times \left(18-Age\right)-0.543\times ln{~}^{18}\!\left/ \!\!{~}_{Age}\right.-0.00763\times \left({18}^{2}-{Age}^{2}\right)+0.0000790\times ({18}^{3}-{Age}^{3})$ in males, and $\mathit{ln}\left(Q\right)=\mathit{ln}\left(SCr\right)+0.177\times \left(18-Age\right)-0.223\times ln{~}^{18}\!\left/ \!\!{~}_{Age}\right.-0.00596\times \left({18}^{2}-{Age}^{2}\right)+0.0000686\times \left({18}^{3}-{Age}^{3}\right)$; *max*(*Age*;18) represents the maximum of *Age* (actual age of the patient) and 18 years (the applied age threshold)
CKD-EPI40 [28]	$eGFR=144\times {({~}^{Q}\!\left/ \!\!{~}_{62}\right.)}^{-0.329}\times {0.993}^{max(Age;40)}$ in females with *Q* ≤ 62 µmol/L, $eGFR=144\times {({~}^{Q}\!\left/ \!\!{~}_{62}\right.)}^{-1.209}\times {0.993}^{max(Age;40)}$ in females with *Q* > 62 µmol/L, $eGFR=141\times {({~}^{Q}\!\left/ \!\!{~}_{80}\right.)}^{-0.411}\times {0.993}^{max(Age;40)}$ in males with *Q* ≤ 80 µmol/L, and $eGFR=141\times {({~}^{Q}\!\left/ \!\!{~}_{80}\right.)}^{-1.209}\times {0.993}^{max(Age;40)}$ in males with *Q* > 80 µmol/L, where *Q* represents age-adjusted creatinine (age 2–39) or actual creatinine (age ≥ 40) and *max*(*Age*;40) represents the maximum of actual age and 40 years. *Q* is obtained from $\mathit{ln}\left(Q\right)=\mathit{ln}\left(SCr\right)+0.259\times \left(40-Age\right)-0.543\times ln{~}^{40}\!\left/ \!\!{~}_{Age}\right.-0.00763\times \left({40}^{2}-{Age}^{2}\right)+0.0000790\times ({40}^{3}-{Age}^{3})$ in males, and $\mathit{ln}\left(Q\right)=\mathit{ln}\left(SCr\right)+0.177\times \left(40-Age\right)-0.223\times ln{~}^{40}\!\left/ \!\!{~}_{Age}\right.-0.00596\times \left({40}^{2}-{Age}^{2}\right)+0.0000686\times \left({40}^{3}-{Age}^{3}\right)$ in females
iCARE [29]	$eGFR=50.7\times {BSA}^{0.816}\times {({~}^{L}\!\left/ \!\!{~}_{SCr}\right.)}^{0.405}\times (0.8994$, if sex = female, | 1 otherwise); in which *L* represents height in cm, *SCr* in µmol/L

Abbreviations:* FAS*, full-age spectrum; *EKFC*, European Kidney Function Consortium; *CKiD*, Chronic Kidney Disease in Children; *CKiDU25*, CKiD under 25; *LMR18*, revised Lund-Malmö extended to children; *CKD-EPI40*, Chronic Kidney Disease Epidemiology Collaboration extended to children; *iCARE*, Improving renal complications in Adolescents with T2D through Research; *SCr*, serum creatinine; *BSA*, body surface area

### Statistical analysis

The basic features of the data in this study were described using descriptive statistics. Summary statistics are presented as mean ± standard deviation (for normally distributed data) and median (interquartile range) otherwise. Minimum, maximum, and percentiles (P5, P25, P75, and P95) are presented for SCr/Q. Distributions of SCr/Q and eGFR predictions are presented as histograms and quantile regression is used to investigate the age-dependency of SCr/Q and eGFR predictions. Generalized linear models were used for SCr/Q and all GFR-estimating equations with age (2-year subgroups), sex, IOTF, and their interactions. All statistics were performed with SAS 9.4 (SAS Institute Inc., Cary, NC, USA). Clinical trial registration: ClinicalTrial.gov; Registration Number: NCT02091544.

## Results

### Characteristics

Six hundred children were included in this study of whom 53.5% were female. Mean age was 12.20 ± 3.28 years. Mean BMI z-score was 3.31 ± 0.75 and the prevalence of overweight, obesity, and severe obesity was 21.3%, 44.7%, and 34.0%, respectively. Mean BSA was 1.78 ± 0.43 m^2^.

### Creatinine values for age

**Fig. 1:**
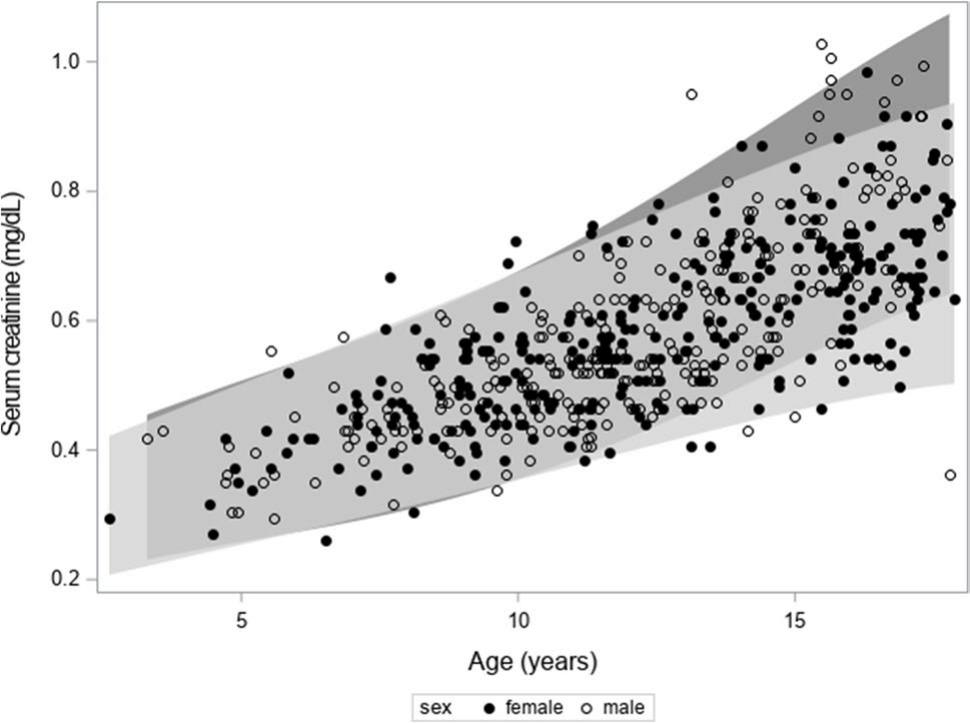
Serum creatinine (SCr, in mg/dL) against age for males and females. The band represents the reference interval for SCr based on the Hoste polynomials [20]

**Fig. 2:**
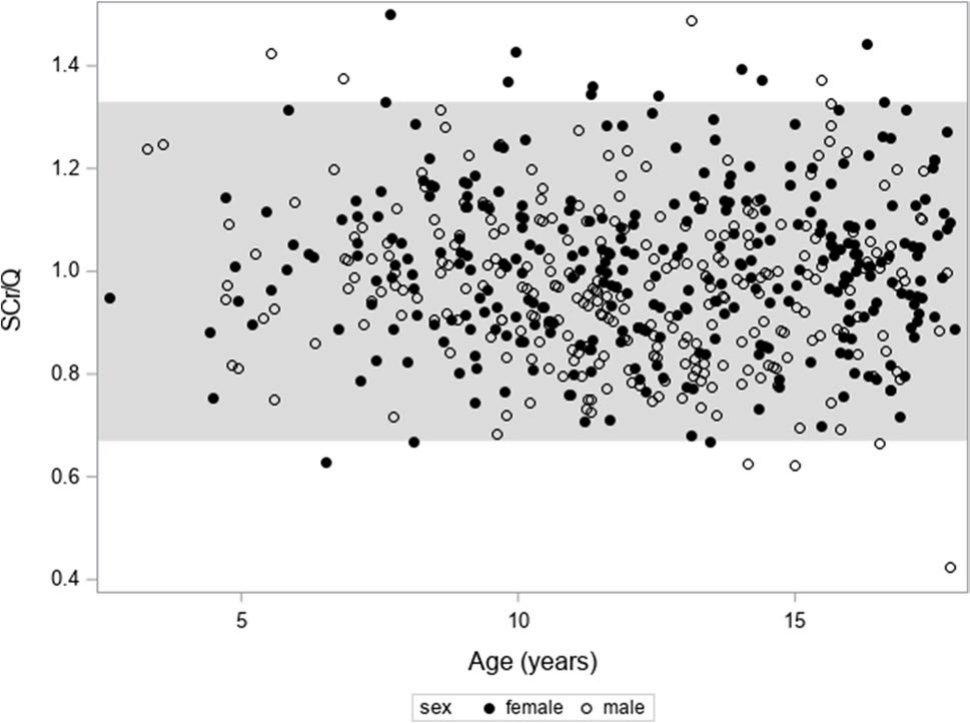
SCr/Q against age with the reference band [0.67–1.33] as described by Pottel et al. [21]. 96.5% of the children have a SCr/Q within this reference band

**Fig. 3:**
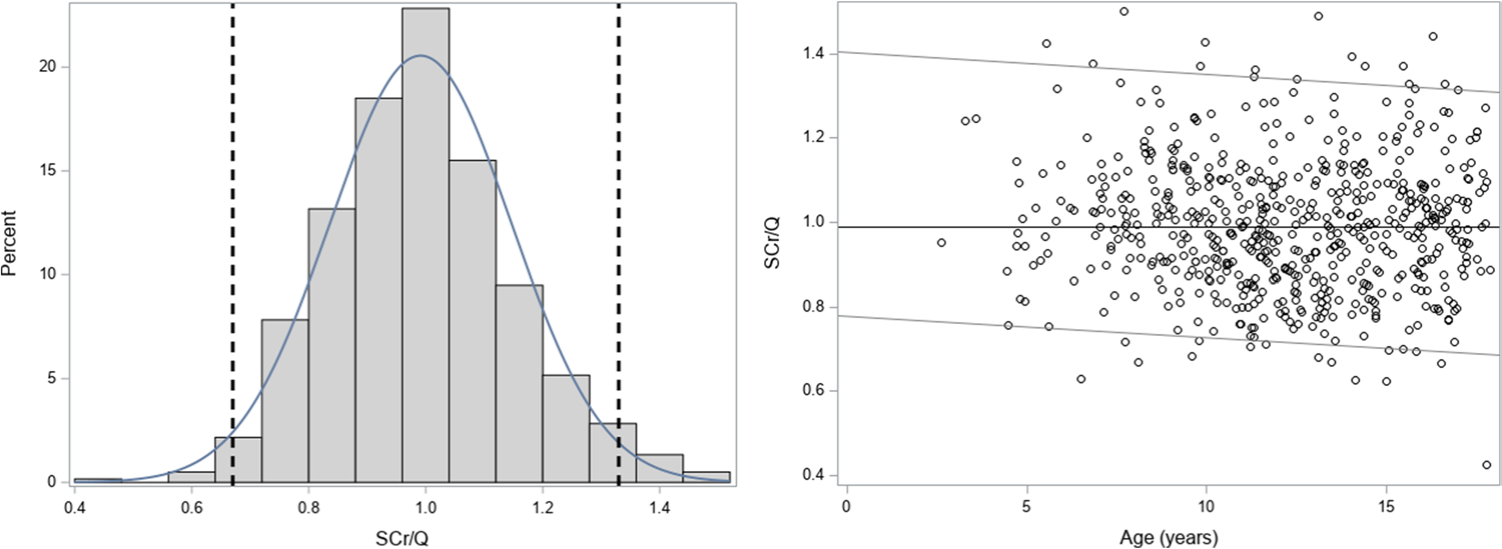
Histogram with normal density function overlaid for normalized or rescaled SCr. The vertical reference lines correspond with the lower and upper limits of 0.67 and 1.33 (left). Linear quantile regression lines for percentiles 2.5, 50, and 97.5 are shown (right)

In Fig. 1, SCr is plotted against age for males and females, in which the band represents the reference interval for SCr based on the Hoste polynomials [20]. From Fig. 1, it can be concluded that most of the children have SCr values lying within the reference band. Moreover, there is a slight difference between the bands for males and females, especially for ages > 15 years. In Fig. 2, SCr/Q (“normalized SCr”) is plotted against age with the reference band [0.67–1.33] as described by Pottel et al. [21]. 96.5% of the children have a SCr/Q within this reference band. There are some children with SCr/Q > 1.33, but they all have SCr/Q ≤ 1.5. There is 1 child with a very low SCr/Q value (a boy aged 17 years and 9 months with a SCr/Q value of 0.43). Normalizing SCr with the Q-values results in data presented in Table 2. Except for the [0–4] year age group (with only 3 subjects), the median and mean SCr/Q is close to “1” for all age groups. In general, there is a small but significant difference between males and females (0.97 vs. 1.01) in mean normalized SCr (*p* = 0.0006). As the standard deviation in each subgroup is stable, it can be expected that the SCr/Q values are normally distributed, which is shown in Fig. 3. The linear quantile regression in the same figure shows a flat median quantile line close to “1.”

**Table 2:** Normalized SCr with the Q-values, stratified per age category

Age (years)	*n*	SCr/Q								
		Mean ± SD	Minimum	P5	P25	Median	P75	P95	Maximum	Interquartile range
[0–4]	3	1.15 ± 0.17	0.95	0.95	0.95	1.24	1.25	1.25	1.25	0.30
[4–6]	22	1.00 ± 0.17	0.75	0.76	0.90	0.97	1.09	1.32	1.42	0.20
[6–8]	45	1.02 ± 0.15	0.63	0.79	0.94	1.02	1.07	1.33	1.50	0.12
[8–10]	84	1.03 ± 0.16	0.67	0.77	0.91	1.02	1.14	1.28	1.43	0.23
[10–12]	136	0.97 ± 0.14	0.71	0.75	0.87	0.96	1.06	1.24	1.36	0.18
[12–14]	109	0.96 ± 0.16	0.67	0.75	0.84	0.94	1.07	1.24	1.49	0.23
[14–16]	112	0.99 ± 0.16	0.62	0.73	0.88	0.99	1.09	1.29	1.39	0.22
[16–18]	89	1.00 ± 0.16	0.43	0.77	0.91	1.01	1.09	1.26	1.44	0.17

### Creatinine-based GFR-estimating equations

**Fig. 4:**
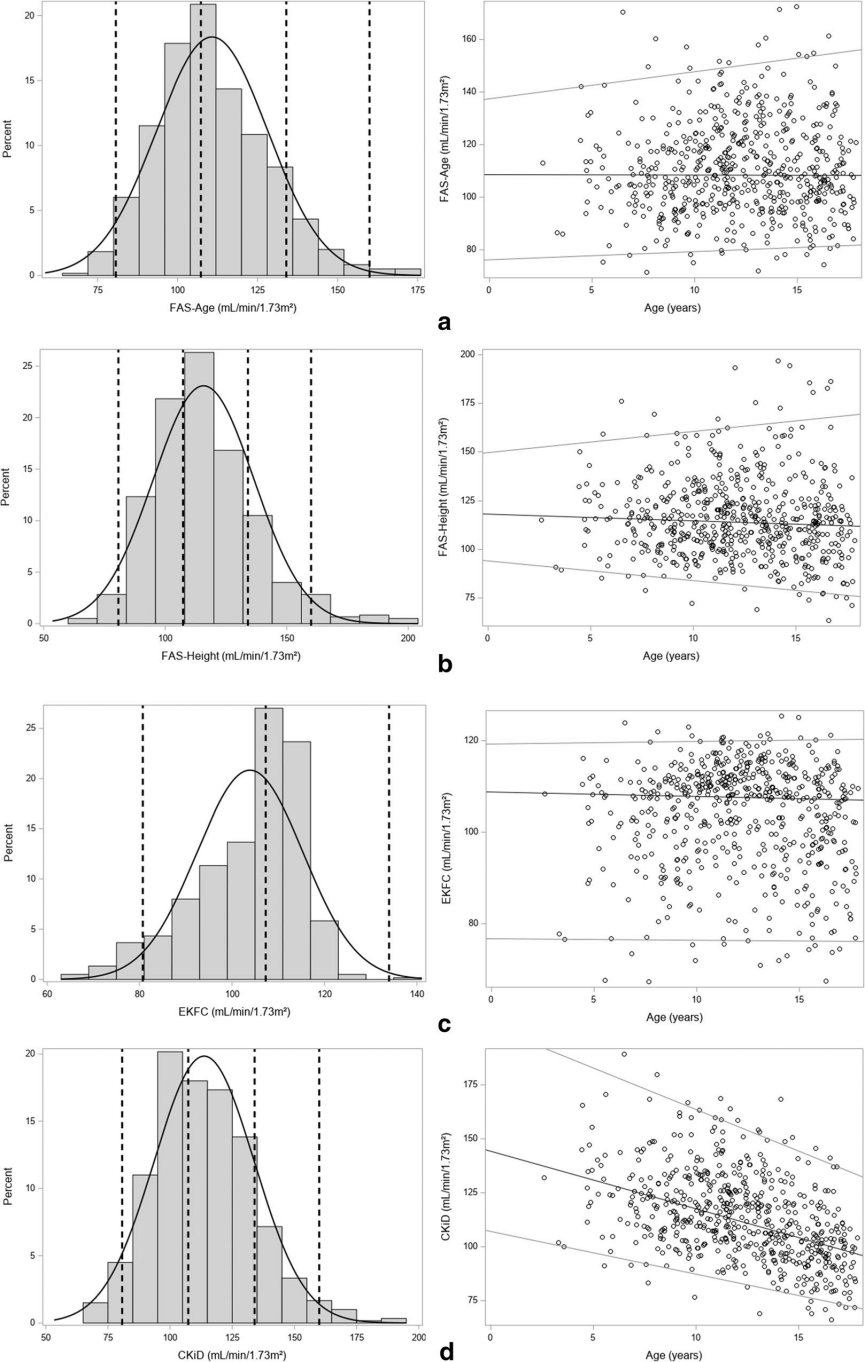

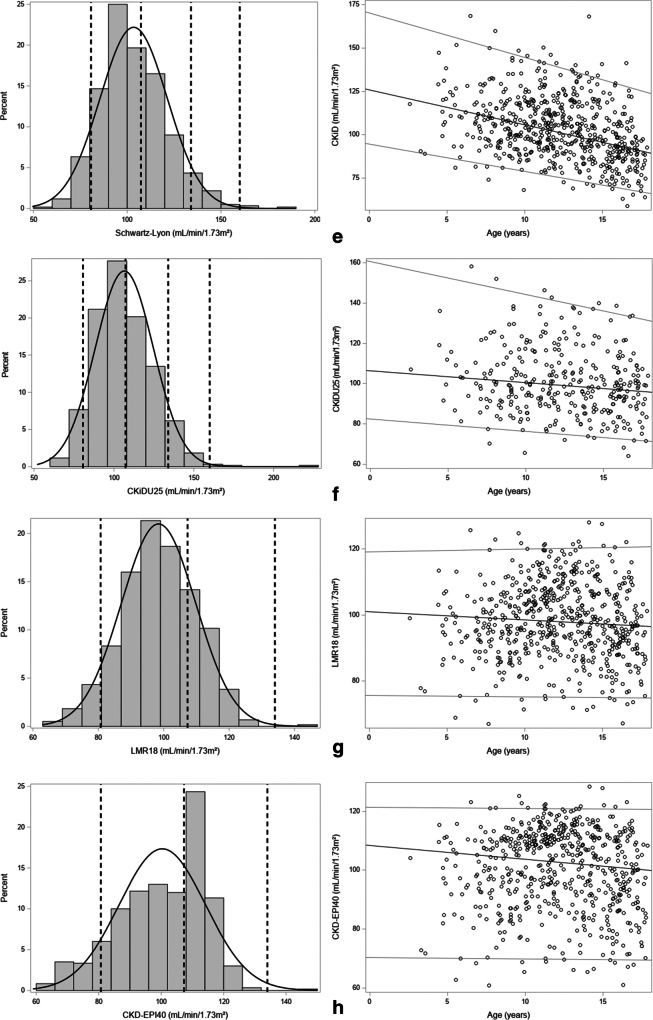
Histograms and linear quantile regression of eGFR FAS age (**a**); FAS height (**b**); EKFC (**c**); CKiD (bedside Schwartz) (**d**); Schwartz-Lyon (**e**); CKiDU25 (**f**); LMR18 (**g**); CKD-EPI40 (**h**). The vertical lines in the histograms correspond to 80.7 (lower limit), 107.3 (median), 133.9 (= symmetrical to 80.7), and 160.1 (upper limit) corresponding to the FAS-eGFR limits calculated from SCr/Q = 0.67, 1, and 1.33 as defined by Pottel et al. [21]

Knowing that the SCr values are within the reference range for age and sex for corresponding healthy children, it would be expected that eGFR is age- and sex-independent. In Fig. 4, histograms and linear quantile regression lines for percentiles 2.5, 50, and 97.5 are presented for the creatinine-based GFR-estimating equations.

#### eGFR FAS age (Fig. 4a)

It is important to note that when SCr/Q is normally distributed, and FAS age was defined as 107.3/(SCr/Q), thus inversely related to SCr/Q, then it is mathematically impossible that FAS age is normally distributed. The distribution will be skewed to the right. However, in the literature, it has never been shown that measured GFR shows a distribution skewed to the right. Therefore, this is an artifact of the FAS equation, and this artifact is especially pronounced when SCr/Q is very low. The outlier of 0.43 can be seen as the outlier of 107.3/0.43 = 250 mL/min/1.73 m^2^ (as mentioned above). A mean of SCr/Q = 1 is expected ideally, which corresponds to FAS age = 107.3 mL/min/1.73 m^2^. The vertical lines correspond to 107.3/1.33 = 80.67 mL/min/1.73 m^2^, that is, the lower limit for FAS age corresponding to the upper limit of 1.33 for SCr/Q. Symmetrical to the lower limit of 80.67 mL/min/1.73 m^2^, 134 mL/min/1.73 m^2^ was defined as the (possible) symmetrical upper limit for FAS age. However, when SCr/Q = 0.67 (the lower limit for rescaled creatinine) was used, then an upper limit for FAS age of 160 mL/min/1.73 m^2^ was found. There is a small but significant difference in eGFR between males and females (113.8 vs. 108.6 mL/min/1.73 m^2^, *p* = 0.0006).

#### eGFR FAS height (Fig. 4b)

Median values are shifted to higher values, compared with FAS age, while the extreme outlier is less outlying. This means that higher Q-values are used in the FAS height equation compared to the FAS age equation. There is a small but significant difference in eGFR between males and females (118.6 vs. 113.4 mL/min/1.73 m^2^, *p* = 0.0021).

#### eGFR EKFC (Fig. 4c)

The EKFC equation is a modified (or optimized) FAS equation, which addresses the flaw of the FAS equation of large overestimation when SCr/Q values are low. Median values may not be affected, but the high eGFR predictions by FAS will not be present when EKFC is used. The distribution is narrower and appears to be skewed to the left (as a consequence of the new power coefficients in the EKFC equation). There is a significant difference in eGFR between males and females (106.8 vs. 101.4 mL/min/1.73 m^2^, *p* < 0.0001).

#### eGFR bedside Schwartz or CKiD (Fig. 4d)

The histogram is relatively symmetrical, although slightly skewed to the right. The age-dependent decline is probably an artifact of the Schwartz-equation in children without CKD, due to using a fixed *k* value of 0.413 throughout the entire age range of 2–18 years. This age-dependency of the CKiD (and the Schwartz-Lyon) equation is not unique to our population, as it can also be observed when applying metadata from healthy children in Belgium [1] to these equations (Supplementary Information). This decline with age is even more pronounced in male adolescents. There is no significant difference in eGFR between females and males (112.4 vs. 115.2 mL/min/1.73 m^2^, *p* = 0.0858).

#### eGFR Schwartz-Lyon (Fig. 4e)

This equation is very similar to the CKiD equation, but differs for females and pre-pubertal males. The same decrease in eGFR is observed as for the eGFR bedside Schwartz equation. Median eGFR value is about 125 mL/min/1.73 m^2^ at 2 years of age and decreases to about 90 mL/min/1.73 m^2^ at 18 years. There is a significant difference in eGFR between males and females (107.2 vs. 100.1 mL/min/1.73 m^2^, *p* < 0.0001).

#### eGFR CKiDU25 (Fig. 4f)

This recently updated CKiD equation no longer showed the decline with age, as a consequence of using age- and sex-dependent *k* values. None of the slopes of the quantile lines was significantly different than zero, when studied separately in males and females. In other words, the CKiDU25 equation does not show an age-dependency in our cohort. However, there is a large difference in eGFR between males and females across the entire age spectrum: the mean eGFR is 100.8 mL/min/1.73 m^2^ for females and 113.2 mL/min/1.73 m^2^ for males (*p* < 0.0001). eGFR values using this CKiDU25 are lower compared to the CKiD equation, especially in children below 10 years of age.

#### eGFR LMR18 (Fig. 4g)

The histogram is relatively symmetrical and the linear quantile regression lines are flat, indicating that there is no age-dependency. There is a significant difference in eGFR between males and females (101.9 vs. 95.4 mL/min/1.73 m^2^, *p* < 0.0001).

#### eGFR CKD-EPI40 for children (Fig. 4h)

This modified CKD-EPI equation does not show an age-dependency, but the distribution of eGFR values is not symmetrical. There is a significant difference in eGFR between males and females (104.7 vs. 96.6 mL/min/1.73 m^2^, *p* < 0.0001).

#### eGFR iCARE

As a result of the BSA correction in this equation, eGFR iCARE increases with age which stabilizes after age 12. In other words, iCARE cannot be used in children aged 12 years and younger, but seems applicable for adolescents with overweight and obesity. It is remarkable that iCARE predicts really high eGFR values for the adolescent group (which it is designed for). No other equation predicts values this high. There is a significant difference in eGFR between males and females (127.7 vs. 113.4 mL/min/1.73 m^2^, *p* < 0.0001).

### Age- and sex-dependency of the creatinine-based GFR-estimating equations

When comparing the young age group (12 years and younger) with the older age group (older than 12 years) in females and males, there was no consistency in the results of the different equations. Briefly, we observed a strong age-decline in CKiD and Schwartz-Lyon, but not in the other equations. Most equations showed small differences between sexes, but CKiD and Schwartz-Lyon show no differences between males and females in young children, but substantial differences in adolescents. CKiDU25 shows systematic differences between males and females over the entire age range. Detailed analyses of the age- and sex-dependency of the creatinine-based GFR-estimating equations can be found in the Supplementary Information.

### Creatinine-based GFR-estimating equations and IOTF criteria

In order to examine possible effects of the different IOTF classes (overweight, obesity, and severe obesity) on SCr/Q and the different SCr-based GFR-estimating equations, generalized linear models were made for SCr/Q and all GFR-estimating equations (as the dependent variable) with age (2-year subgroups), sex, IOTF, and their interactions (as independent variables). SCr/Q was not affected by age and sex (as supposed), nor by IOTF and their interactions. The height-dependent equations (FAS height, CKiD, Schwartz-Lyon, and CKiDU25) demonstrated a minor but significant IOTF dependency, with slightly higher eGFR values in children with obesity and severe obesity compared to children with overweight. The age-dependent equations (FAS age, EKFC, LMR18, and CKD-EPI40) did not show an IOTF dependency.

## Discussion

The main message from this study is that serum creatinine (SCr) values of children with overweight and obesity were mostly within the reference range. Q-age polynomials (the median SCr for healthy children and adolescents) from reference values [20] can be used to normalize serum creatinine (“SCr/Q”) in our cohort, resulting in a value that is age- and sex-independent, evidenced by a median and mean SCr/Q close to “1” for all age groups. As 96.5% of the children with overweight and obesity in this study have a SCr/Q within the reference interval [0.67–1.33] [21], this reference interval can be used to evaluate the normality (or abnormality) of normalized SCr in children with overweight and obesity without overt kidney disease.

Creatinine is the most commonly used endogenous marker for kidney function in children and adults [3]. Until approximately two decades ago, reference values for SCr varied between hospitals. Since the implementation of isotope dilution mass spectroscopy (IDMS)-based calibration of creatinine measurement, reference values for SCr in the pediatric population are uniform over the entire age spectrum [19, 30]. Normal values for SCr are heavily age-dependent, as SCr increases with muscle mass and (thus) age, with the exception of the neonatal period. Until the age of 14 years, there is no sex-specific difference in SCr, while from the age of 14 years, normal values of SCr in male adolescents are higher than in females [19]. Children with overweight and obesity are taller and have an increased absolute fat mass (FM) and fat-free mass (FFM) compared to age- and sex-matched normal weight children; approximately 75% of the excess weight is FM, the remainder FFM [31]. Based on this, one should expect higher SCr values in children with overweight and obesity compared to lean children. We here showed that SCr values of children with overweight and obesity are within the reference range. While some studies have demonstrated similar SCr values between children with lean weight and overweight or obesity [32–35], in other studies [36, 37], SCr values were higher in children with overweight or obesity compared to lean controls. It is not excluded that these inconsistent results are due to confounding factors such as age, height, and sex and/or due to the heterogeneity of the methods for SCr measurements. Finally, SCr reference values are derived from healthy individuals [19, 30]. In both reference studies, anthropometry or body proportion was not mentioned, and therefore, the prevalence of overweight or obesity is not known. It is therefore conceivable that SCr reference values are different, possibly lower, in a cohort of exclusively normal weight children.

Since SCr in our cohort is within the reference range for children, and we did not observe an age- and sex-dependency of SCr, it is not expected to see abnormal kidney function; hence, one can expect normal GFR (in ml/min/1.73 m^2^) and no age/sex-dependency. Also, it is known that measured GFR is stable between 2 and 40 years of age, around 100–110 mL/min/1.73 m^2^. Combining these 2 arguments, it was expected to see a flat and sex-independent GFR age relationship for the median and for the quantiles. This is clearly not the case for the CKiD (bedside Schwartz) [24] and Schwartz-Lyon equation [25] which show a decreasing eGFR age pattern. This age-dependency of the CKiD and Schwartz-Lyon equation is not unique to our population, as it can also be observed when applying metadata from healthy children in Belgium [1] to these equations (Supplementary Information). It can therefore be concluded that these equations, that were developed using data from growth-retarded children with CKD, are not suitable for children of normal height without CKD, with and without overweight and (severe) obesity. The CKiDU25 equation [26] overcomes the age-dependency by using age- and sex-dependent *k* values. However, in our cohort, this equation exhibits a large difference in eGFR between males and females over the entire age spectrum. This sex difference deviates from what is observed with other equations and from what is observed in healthy males and females. On the other hand, the iCARE equation [29] shows an increasing eGFR age pattern. As a result of the BSA correction in this equation, eGFR iCARE increases with age which stabilizes after age 12. In other words, iCARE cannot be used in children 12 years old and younger, but seems applicable for adolescents with overweight and obesity. However, it should be mentioned that iCARE predicts very different values for adolescent males and females (a difference of 15 mL/min/1.73 m^2^) and 25–30% higher values compared to the other equations. It should be noted that the iCARE equation was designed for children with overweight/obesity and type 2 diabetes mellitus, but these large differences require further investigation and the need for measured GFR. FAS age [22], FAS height [20], and LMR18 [27] equations show a relatively symmetrical distribution and no age-dependency. The EKFC [23] and CKD-EPI40 formulas [28] also do not show an age-dependency, but the distribution of eGFR values is not symmetrical. There is no consistency between the equations regarding the differences between males and females in young and adolescent age groups. Finally, the height-dependent equations (FAS height, CKiD, Schwartz-Lyon, and CKiDU25) indicate a minor but statistically significant higher eGFR in children with obesity and severe obesity, compared to children with overweight. The age-dependent equations (FAS age, EKFC, LMR18, and CKD-EPI) do not show such a dependency. Considering all of this, we suggest that eGFR FAS age, FAS height, and LMR18 can be allotted as the preferred eGFR equations in children with overweight and obesity.

This study is not without limitations. First and most important, it lacks a gold standard measurement of GFR. It is therefore impossible to state whether the preferred creatinine-based GFR-estimating equation can reflect real kidney function in children with overweight and obesity. In adults with overweight and obesity, the random error of creatinine-based GFR-estimating equations is wide; 90% of the estimations had an error within ± 55% of measured GFR, determined using plasma iohexol disappearance [2]. Another limitation of this study is the lack of another endogenous marker for kidney function, such as cystatin C. Where creatinine heavily depends on muscle mass, this does not apply to cystatin C [38]. However, cystatin C synthesis is upregulated in adipose tissue in case of obesity [39] and cystatin C does not improve reliability of GFR estimation over SCr in adults with overweight and obesity [2]. However, there are studies in children in which cystatin C improves the predictive performance of eGFR equations [3]. Finally, an approach in which SCr- and cystatin C-based eGFR are compared, instead of combined into more complex equations, can add confidence to the accuracy of the GFR [40] and can have additional advantages as well [3].

Therefore, we suggest that future research implement other endogenous markers for kidney function, such as cystatin C and measured GFR in order to examine the role of GFR-estimating equations in children with overweight and obesity. It is possible that such studies could also contribute to our understanding of whether GFR-indexation with BSA, or de-indexation of eGFR is helpful in children with overweight and obesity, a topic that is controversial in individuals with altered body proportions [12, 41].

In conclusion, SCr values of children with overweight and obesity without overt kidney disease are mostly within the reference range. Normalization of SCr using reference Q-age polynomials works very well in this cohort. After evaluation of the different equations, we suggest that FAS age, FAS height, and LMR18 are the preferred creatinine-based GFR-estimating equations in children with overweight and obesity.

## Supplementary Information

Below is the link to the electronic supplementary material.

Supplementary file1 (DOCX 44 KB)

Supplementary file2 (pptx 134 KB)

## Data Availability

The datasets generated during and/or analyzed during the current study are available from the corresponding author on reasonable request due to privacy and other restrictions.

## Abbreviations

*SCr/Q*: Serum creatinine normalized using the Q-age polynomials
*n*: Number of children
*P*: Percentile
