# Supplementary material for: Creatinine-based GFR-estimating equations in children with overweight and obesity
Source: Pediatr Nephrol. 2022 Feb 24;37(10):2393–403. doi: 10.1007/s00467-021-05396-y (PMC9395456; doi:10.1007/s00467-021-05396-y)
Supplement: Supplementary file 1 — Supplementary file1 (DOCX 44 KB) [file 467_2021_5396_MOESM1_ESM.docx]

**Supplementary Information**

**Creatinine-based GFR-estimating equations in children with overweight and obesity**

*Age-dependency of the creatinine-based GFR-estimating equations*

In order to evaluate the age dependency in more detail, we used 2 age groups: a) ≤ 12 years and b) > 12 years and calculated descriptive statistics for both age groups, displayed in Supplementary Table 1. For FAS age, FAS height, EKFC, CKiDU25, LMR18 and CKD-EPI40, the difference between the mean (or median) values is small, mostly less than 2-3 mL/min/1.73m². However, this is clearly different for the CKiD (bedside Schwartz), Schwartz-Lyon and iCARE equations where large differences (10-15 mL/min/1.73m² for CKiD and close to 30 mL/min/1.73m² for iCARE) between the means (or medians) of the age groups are observed, with a lower mean value for the Schwartz equations in the > 12-year age group, but contrary, with a higher mean value for iCARE, compared to the ≤ 12-year age group. For iCARE, we believe that this equation should not be used in the ≤ 12-year age group.

**Table 1.** Descriptive statistics of the different creatinine-based GFR-estimating equations stratified according to age group ≤ 12 years and > 12 years

| **Age group** | **n** | **Equations** | **Mean ± SD** | **P5** | **P25** | **P50** | **P75** | **P95** | **Quartile range** |
| --- | --- | --- | --- | --- | --- | --- | --- | --- | --- |
| ≤ 12 years | 290 | FAS-age | 109.9 ± 16.6 | 84.1 | 97.7 | 108.4 | 118.7 | 142.0 | 21.0 |
|  |  | FAS-height | 116.3 ± 18.2 | 90.2 | 103.3 | 115.2 | 126.7 | 151.3 | 23.4 |
|  |  | EKFC | 104.5 ± 10.8 | 81.3 | 98.8 | 108.1 | 111.6 | 117.3 | 12.8 |
|  |  | CKiD | 121.2 ± 18.6 | 94.0 | 107.7 | 119.6 | 132.6 | 155.2 | 24.9 |
|  |  | Schwartz-Lyon | 108.0 ± 16.6 | 83.8 | 96.0 | 106.6 | 118.2 | 138.3 | 22.2 |
|  |  | CKiDU25 | 107.9 ± 17.0 | 82.5 | 94.9 | 105.9 | 119.3 | 138.5 | 24.4 |
|  |  | LMR18 | 98.8 ± 10.7 | 80.6 | 92.2 | 99.0 | 105.9 | 116.0 | 13.8 |
|  |  | CKD-EPI40 | 101.1 ± 13.0 | 76.3 | 92.7 | 104.3 | 110.7 | 117.8 | 18.0 |
|  |  | iCARE | 105.5 ± 19.7 | 75.9 | 91.6 | 104.6 | 119.5 | 139.4 | 28.0 |
| > 12 years | 310 | FAS-age | 112.1 ± 19.8 | 84.8 | 98.6 | 108.6 | 123.3 | 142.4 | 24.7 |
|  |  | FAS-height | 115.3 ± 22.8 | 83.3 | 100.9 | 113.4 | 124.4 | 158.3 | 23.6 |
|  |  | EKFC | 103.3 ± 12.1 | 78.6 | 96.0 | 107.3 | 112.1 | 117.7 | 16.1 |
|  |  | CKiD | 106.7 ± 18.9 | 79.3 | 93.6 | 104.0 | 118.5 | 140.0 | 24.9 |
|  |  | Schwartz-Lyon | 99.1 ± 18.2 | 72.6 | 86.4 | 96.4 | 109.6 | 131.0 | 23.1 |
|  |  | CKiDU25 | 105.3 ± 19.2 | 77.8 | 93.0 | 102.9 | 117.7 | 136.2 | 24.8 |
|  |  | LMR18 | 98.0 ± 12.0 | 77.0 | 90.0 | 97.3 | 107.0 | 116.5 | 17.1 |
|  |  | CKD-EPI40 | 99.6 ± 14.5 | 72.0 | 89.2 | 101.4 | 111.3 | 118.2 | 22.1 |
|  |  | iCARE | 133.7 ± 19.5 | 104.6 | 121.3 | 132.0 | 144.5 | 165.0 | 23.2 |

Note that iCARE should not be used in children ≤ 12 years of age (as it was designed for adolescents). Abbreviations: n, number of children; P, percentile; FAS, Full-Age Spectrum; EKFC, European Kidney Function Consortium; CKiD, Chronic Kidney Disease in Children; CKiDU25, CKiD under 25; LMR18, revised Lund-Malmö; CKD-EPI, Chronic Kidney Disease Epidemiology Collaboration; iCARE, Improving renal complications in Adolescents with T2D through Research; SCr, serum creatinine

*Age-dependency of the CKiD and iCARE equations using metadata*

In order to examine whether the age-dependency of the CKiD and iCARE is unique to our population, metadata from healthy children in Belgium [1] were applied to these equations. It is clear that eGFR CKiD decreases with age, which is not expected in healthy children, nor in children with overweight and obesity (since SCr are comparable with normal reference values). This age-dependency is more outspoken in male adolescents, and is the result of the age- and sex-independent fixed k value of 0.413. The eGFR CKiDU25, that uses an age and sex-dependent k value, does not show this age dependency. However, the CKiDU25 shows a remarkable sex difference. Since this topic is beyond the scope of this article, the interested reader is referred to a forthcoming article.

In Supplementary Figure 1, eGFR iCARE is plotted against age. It is clear that eGFR iCARE increases with age which stabilizes in adolescents. In other words, iCARE cannot be used in the full-age spectrum in children, but seems applicable for adolescents.

**Supplementary Figure 1.** eGFR-iCARE (ml/min/1.73m^2^) plotted against age (years) when applying metadata from healthy children in Belgium [1] to this equation

*Sex differences between the creatinine-based GFR-estimating equations*

We analysed sex differences using the same age groups, as displayed in Supplementary Table 2 (for children ≤ 12 years) and Supplementary Table 3 (for children > 12 years). SCr/Q is equal to 1.01 (female ≤ 12 years), 0.99 (female > 12 years), 1.01 (male ≤ 12 years), and 0.95 (male > 12 years), respectively. As these differences are small, this is reflected in small differences of mean FAS age: 108.9, 110.9, 108.4 and 116.8 mL/min/1.73m², and in FAS height: 115.3, 117.3, 111.7 and 119.9 mL/min/1.73m². Values are slightly higher for eGFR in males ≤ 12 years than in females ≤ 12 years, and mildly higher in male adolescents. Note that the Q values for FAS do not differ between males and females in the young age group [1], Therefore, it is not expected to see large differences between females and males in this age group. LMR18 and CKD-EPI40 show higher values (around 5-7 mL/min/1.73m²) in males than in females, but no real difference between the young and adolescent age groups. The CKiD (bedside Schwartz) equation shows a totally different picture: no real difference between males and females within the same age group, but higher in the young age group (around 120 mL/min/1.73m²) than in the adolescent age group (around 105-108 mL/min/1.73m²). Schwartz-Lyon shows no difference between males and females in the young age group but a large difference in the adolescent age group (around 11 mL/min/1.73m²). Note that Schwartz-Lyon uses different k values in adolescent females and males, which may explain the difference. CKiDU25 shows large differences between males and females (around 11 mL/min/1.73m²) in both age groups, but much smaller differences between sex-specific age groups. iCARE (if only considering the > 12-year age group) shows a clear difference between females and males, 126.5 and 143 ml/min/1.73m^2^, respectively. Compared to the other equations, iCARE shows significantly higher eGFR-predictions, which is probably the effect of the BSA correction in this equation.

**Table 2.** Descriptive statistics of the different creatinine-based GFR-estimating equations stratified according to sex for males and females of 12 years and younger

| **Sex** | **n** | **Variables** | **Mean ± SD** | **P5** | **P25** | **P50** | **P75** | **P95** | **Quartile range** |
| --- | --- | --- | --- | --- | --- | --- | --- | --- | --- |
| Female | 146 | SCr/Q | 1.01 ± 0.16 | 0.76 | 0.90 | 1.00 | 1.11 | 1.29 | 0.21 |
|  |  | FAS-age | 108.9 ± 17.1 | 83.4 | 96.8 | 106.8 | 119.5 | 141.0 | 22.7 |
|  |  | FAS-height | 115.3 ± 19.5 | 89.6 | 101.9 | 111.6 | 126.7 | 151.3 | 24.8 |
|  |  | EKFC | 102.8 ± 11.3 | 80.4 | 94.6 | 106.3 | 110.9 | 117.0 | 16.3 |
|  |  | CKiD | 120.5 ± 19.9 | 91.7 | 106.7 | 117.0 | 133.9 | 159.6 | 27.2 |
|  |  | Schwartz-Lyon | 107.3 ± 17.8 | 81.7 | 95.0 | 104.3 | 119.3 | 142.2 | 24.4 |
|  |  | CKiDU25 | 103.0 ± 16.9 | 79.7 | 91.5 | 100.3 | 113.1 | 136.2 | 21.6 |
|  |  | LMR18 | 96.6 ± 10.7 | 78.5 | 89.0 | 96.3 | 103.9 | 114.8 | 15.0 |
|  |  | CKD-EPI40 | 98.3 ± 13.4 | 73.7 | 87.7 | 99.3 | 110.0 | 116.2 | 22.3 |
|  |  | iCARE | 97.7 ± 17.5 | 72.9 | 84.4 | 96.5 | 108.1 | 128.7 | 23.7 |
| Male | 144 | SCr/Q | 0.99 ± 0.14 | 0.75 | 0.91 | 0.97 | 1.08 | 1.24 | 0.18 |
|  |  | FAS-age | 110.9 ± 16.0 | 86.6 | 98.9 | 110.5 | 118.4 | 142.8 | 19.5 |
|  |  | FAS-height | 117.3 ± 16.9 | 91.7 | 105.5 | 116.4 | 126.7 | 150.3 | 21.2 |
|  |  | EKFC | 106.2 ± 10.1 | 86.2 | 101.8 | 109.3 | 112.2 | 119.6 | 10.4 |
|  |  | CKiD | 122.0 ± 17.2 | 96.0 | 109.5 | 121.7 | 132.3 | 153.5 | 22.9 |
|  |  | Schwartz-Lyon | 108.7 ± 15.3 | 85.6 | 97.5 | 108.4 | 117.9 | 136.8 | 20.4 |
|  |  | CKiDU25 | 112.9 ± 15.7 | 89.4 | 101.5 | 112.5 | 122.0 | 143.6 | 20.5 |
|  |  | LMR18 | 101.1 ± 10.3 | 84.3 | 94.7 | 101.8 | 107.3 | 119.5 | 12.6 |
|  |  | CKD-EPI40 | 104.0 ± 11.9 | 81.4 | 97.3 | 107.7 | 111.5 | 120.8 | 14.2 |
|  |  | iCARE | 113.4 ± 18.7 | 81.5 | 101.4 | 114.9 | 124.0 | 145.1 | 22.6 |

Note that iCARE was not designed for children ≤ 12 years of age. Abbreviations: SCr/Q, serum creatinine normalized using the Q-age polynomials; n, number of children; P, percentile; FAS, Full-Age Spectrum; EKFC, European Kidney Function Consortium; CKiD, Chronic Kidney Disease in Children; CKiDU25, CKiD under 25; LMR18, revised Lund-Malmö; CKD-EPI, Chronic Kidney Disease Epidemiology Collaboration; iCARE, Improving renal complications in Adolescents with T2D through Research; SCr, serum creatinine

**Table 3.** Descriptive statistics of the different creatinine-based GFR-estimating equations stratified according to sex for males and females older than 12 years

| **Sex** | **n** | **Variables** | **Mean ± SD** | **P5** | **P25** | **P50** | **P75** | **P95** | **Quartile range** |
| --- | --- | --- | --- | --- | --- | --- | --- | --- | --- |
| Female | 175 | SCr/Q | 1.01 ± 0.15 | 0.77 | 0.91 | 1.01 | 1.11 | 1.30 | 0.20 |
|  |  | FAS-age | 108.4 ± 16.9 | 82.8 | 96.5 | 105.8 | 117.6 | 138.9 | 21.1 |
|  |  | FAS-height | 111.7 ± 22.0 | 82.1 | 96.4 | 110.5 | 120.1 | 152.9 | 23.7 |
|  |  | EKFC | 100.2 ± 11.9 | 78.0 | 92.4 | 102.2 | 109.5 | 115.9 | 17.1 |
|  |  | CKiD | 105.6 ± 18.2 | 80.4 | 93.4 | 103.5 | 116.3 | 142.0 | 22.8 |
|  |  | Schwartz-Lyon | 94.1 ± 16.2 | 71.6 | 83.2 | 92.2 | 103.6 | 126.5 | 20.3 |
|  |  | CKiDU25 | 98.9 ± 15.8 | 76.6 | 87.5 | 97.7 | 107.3 | 132.7 | 19.8 |
|  |  | LMR18 | 94.3 ± 10.9 | 76.5 | 87.5 | 93.9 | 101.2 | 113.0 | 13.8 |
|  |  | CKD-EPI40 | 95.2 ± 14.0 | 71.3 | 85.5 | 95.3 | 108.2 | 115.1 | 22.7 |
|  |  | iCARE | 126.5 ± 16.0 | 101.5 | 115.2 | 125.5 | 138.0 | 150.7 | 22.8 |
| Male | 135 | SCr/Q | 0.95 ± 0.16 | 0.72 | 0.82 | 0.95 | 1.05 | 1.23 | 0.23 |
|  |  | FAS-age | 116.8 ± 22.1 | 87.5 | 102.1 | 112.9 | 130.6 | 149.0 | 28.5 |
|  |  | FAS-height | 120.0 ± 23.2 | 86.3 | 105.9 | 116.5 | 129.5 | 162.9 | 23.6 |
|  |  | EKFC | 107.4 ± 11.0 | 83.8 | 102.1 | 110.0 | 114.9 | 120.2 | 12.8 |
|  |  | CKiD | 108.0 ± 19.8 | 78.8 | 94.6 | 106.0 | 120.3 | 140.0 | 25.7 |
|  |  | Schwartz-Lyon | 105.7 ± 18.7 | 78.8 | 93.2 | 104.0 | 118.5 | 135.7 | 25.3 |
|  |  | CKiDU25 | 113.6 ± 20.0 | 85.8 | 100.8 | 111.4 | 124.2 | 143.5 | 23.4 |
|  |  | LMR18 | 102.8 ± 11.8 | 82.5 | 94.8 | 102.7 | 111.9 | 120.5 | 17.1 |
|  |  | CKD-EPI40 | 105.4 ± 13.1 | 79.0 | 97.5 | 108.3 | 114.8 | 121.6 | 17.3 |
|  |  | iCARE | 143.0 ± 19.8 | 116.1 | 129.0 | 140.2 | 152.6 | 182.9 | 23.6 |

Abbreviations: SCr/Q, serum creatinine normalized using the Q-age polynomials; n, number of children; P, percentile; FAS, Full-Age Spectrum; EKFC, European Kidney Function Consortium; CKiD, Chronic Kidney Disease in Children; CKiDU25, CKiD under 25; LMR18, revised Lund-Malmö; CKD-EPI, Chronic Kidney Disease Epidemiology Collaboration; iCARE, Improving renal complications in Adolescents with T2D through Research; SCr, serum creatinine

**References**

1. Pottel H (2017) Measuring and estimating glomerular filtration rate in children. Pediatr Nephrol 32:249-263
