# Supplementary material for: Creatinine-based GFR-estimating equations in children with overweight and obesity
Source: Pediatr Nephrol. 2022 Feb 24;37(10):2393–403. doi: 10.1007/s00467-021-05396-y (PMC9395456; doi:10.1007/s00467-021-05396-y)
Supplement: Supplementary file 2 — Supplementary file2 (pptx 134 KB) [file 467_2021_5396_MOESM2_ESM.pptx]

## Slide 1
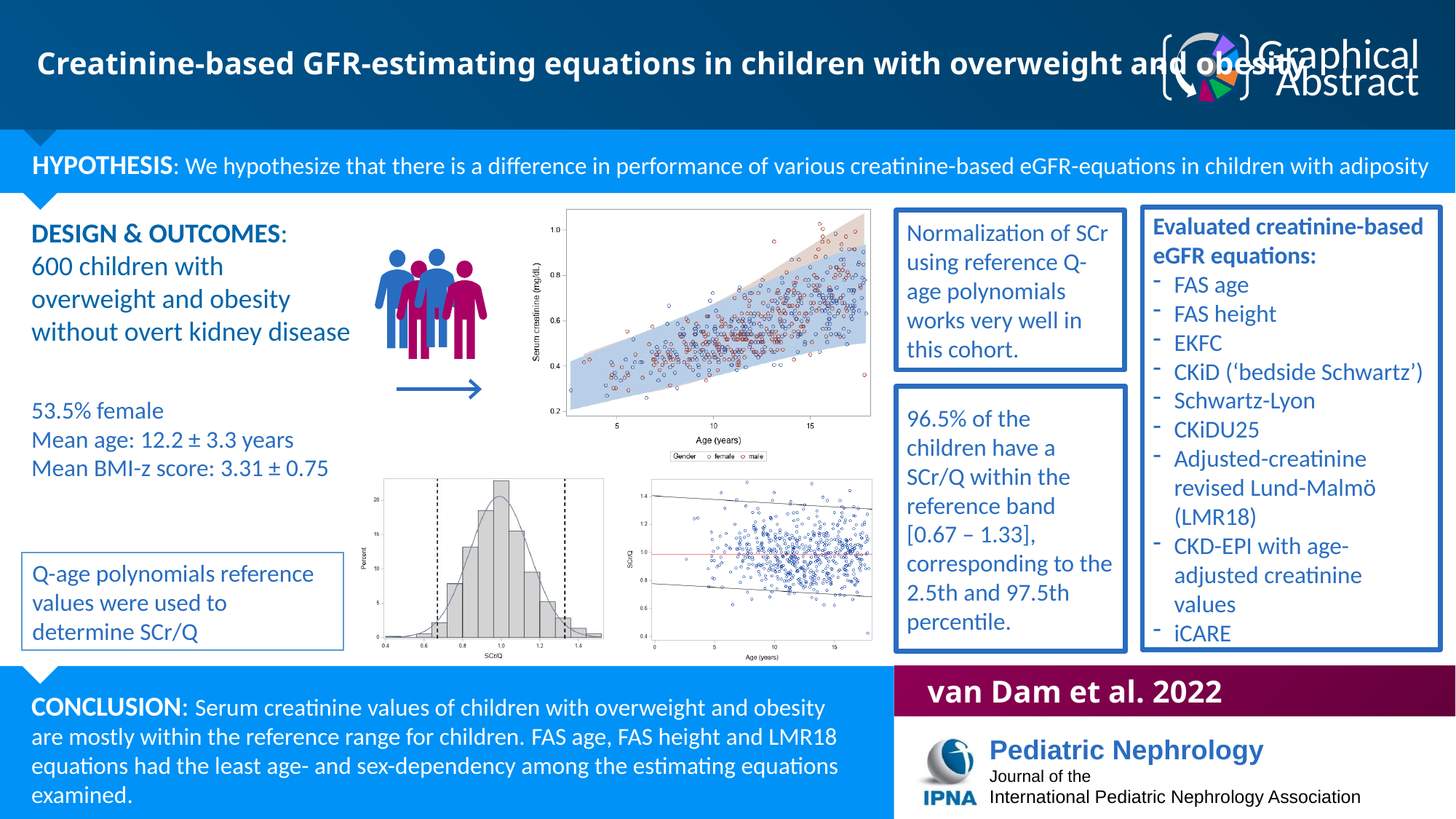

Creatinine-based GFR-estimating equations in children with overweight and obesity
HYPOTHESIS: We hypothesize that there is a difference in performance of various creatinine-based eGFR-equations in children with adiposity
Evaluated creatinine-based eGFR equations:
FAS age
FAS height
EKFC
CKiD (‘bedside Schwartz’)
Schwartz-Lyon
CKiDU25
Adjusted-creatinine revised Lund-Malmö (LMR18)
CKD-EPI with age-adjusted creatinine values
iCARE
DESIGN & OUTCOMES:
600 children with overweight and obesity without overt kidney disease
Normalization of SCr using reference Q-age polynomials works very well in this cohort.
96.5% of the children have a SCr/Q within the reference band
[0.67 – 1.33], corresponding to the 2.5th and 97.5th percentile.
53.5% female
Mean age: 12.2 ± 3.3 years
Mean BMI-z score: 3.31 ± 0.75
Q-age polynomials reference values were used to determine SCr/Q
van Dam et al. 2022
CONCLUSION: Serum creatinine values of children with overweight and obesity are mostly within the reference range for children. FAS age, FAS height and LMR18 equations had the least age- and sex-dependency among the estimating equations examined.
